# Crystal structure of TRIM20 C-terminal coiled-coil/B30.2 fragment: implications for the recognition of higher order oligomers

**DOI:** 10.1038/srep10819

**Published:** 2015-06-04

**Authors:** Christopher Weinert, Damien Morger, Aleksandra Djekic, Markus G. Grütter, Peer R. E. Mittl

**Affiliations:** 1 Department of Biochemistry, University Zürich, Winterthurerstrasse 190, 8057 Zürich, Switzerland

## Abstract

Many tripartite motif-containing (TRIM) proteins, comprising RING-finger, B-Box, and coiled-coil domains, carry additional B30.2 domains on the C-terminus of the TRIM motif and are considered to be pattern recognition receptors involved in the detection of higher order oligomers (e.g. viral capsid proteins). To investigate the spatial architecture of domains in TRIM proteins we determined the crystal structure of the TRIM20Δ413 fragment at 2.4 Å resolution. This structure comprises the central helical scaffold (CHS) and C-terminal B30.2 domains and reveals an anti-parallel arrangement of CHS domains placing the B-box domains 170 Å apart from each other. Small-angle X-ray scattering confirmed that the linker between CHS and B30.2 domains is flexible in solution. The crystal structure suggests an interaction between the B30.2 domain and an extended stretch in the CHS domain, which involves residues that are mutated in the inherited disease Familial Mediterranean Fever. Dimerization of B30.2 domains by means of the CHS domain is crucial for TRIM20 to bind pro-IL-1β *in vitro*. To exemplify how TRIM proteins could be involved in binding higher order oligomers we discuss three possible models for the TRIM5α/HIV-1 capsid interaction assuming different conformations of B30.2 domains.

Tripartite motif-containing proteins represent a large family of proteins sharing a conserved domain architecture. The tripartite motif (TRIM) consists of an N-terminal RING domain, one or two B-box domains and a C-terminal coiled-coil domain. The TRIM segment is considered to serve as a scaffold domain that is extended on either side by additional protein-protein interaction modules. The RING- and B-box domains contain zinc finger motifs that confer E3 ubiquitin ligase and self-association activities, respectively. TRIM proteins are involved in diverse cellular functions including regulation of the innate immune system, retroviral restriction, cell proliferation, and differentiation^1^,^2^.

TRIM20 (Uniprot ID: O15553), which is also named pyrin or marenostrin, is special among other TRIM proteins, because it lacks the RING domain that confers ubiquitin ligase activity. In TRIM20 the RING domain is replaced by a PYD domain, which belongs to the death domain superfamily and is frequently involved in homotypic protein-protein interactions (reviewed in ref 3.). Depending on the splice variant of TRIM20 the PYD domain is connected to the B-box domain by either a 60 or a 280 amino acid long linker. Almost 50% of all TRIM proteins - including TRIM20 - carry a B30.2 domain immediately after the coiled-coil domain of the central TRIM motif (Fig. 1A). The B30.2 domain, which was initially called PrySpry domain, because it consists of an N-terminal Pry- and a C-terminal Spry segment, is involved in heterotypic protein-protein interactions^4^. Although TRIM proteins can be recognized by their characteristic signature sequence, their biological functions are often incompletely understood. Many TRIM proteins act as cytoplasmic pattern recognition receptors that sense higher order oligomers, e.g. a lattice of viral capsid (CA) proteins (reviewed in ref 5.).

TRIM20 is associated with the auto-inflammatory disease Familial Mediterranean Fever (FMF) and it is involved in the modulation of the pro-inflammatory cytokine pro-interleukin-1β (pro-IL-1β)^6^,^7^. The pro-IL-1β/TRIM20 interaction is ambiguous. TRIM20 is considered to inhibit the inflammasome mediated maturation of pro-IL-1β^8^, but it can also potently induce the maturation of this cytokine^9^,^10^,^11^,^12^. The exact mechanism of regulation between these two contrary functions is currently unknown. TRIM5α and TRIM21 are two well-studied examples of TRIM proteins involved in virus recognition. TRIM5α acts as a pattern recognition receptor that confers restriction against HIV-1, HIV-2 and other enveloped viruses in a species-specific manner^13^,^14^,^15^,^16^, whereas TRIM21 recognizes opsonized viruses in the cytoplasm by binding to the Fc-fragment of the antibody^17^,^18^.

TRIM proteins are flexible structural entities that tend to oligomerize upon ligand binding. This behavior makes crystallization of full-length TRIM proteins extremely difficult. Hence, only structures of TRIM protein fragments are known so far. Crystal structures of B30.2 domains from TRIM5α^19^,^20^, TRIM20^21^, TRIM25^22^, TRIM72^23^, and Butyrophilin 3A1^24^ have been determined but only the structures of TRIM21^18^,^25^ and Ret finger protein-like 4A^26^ reveal interactions between B30.2 domains and peptide ligands. The structures of TRIM69 coiled-coil^27^, TRIM25 coiled-coil^28^, and TRIM5α B-box/coiled-coil domains^29^ provide insight into the assembly of the scaffold domain, but the spatial orientation between the coiled-coil and B30.2 domains remains elusive.

Here we present the crystal structure of the TRIM20 coiled-coil/B30.2 fragment at 2.4 Å resolution. This structure shows the overall architecture of TRIM proteins and suggests two distinct spatial orientations between B30.2 and coiled-coil domains. The flexibility between the coiled-coil and B30.2 domains in solution was confirmed by small-angle X-ray scattering (SAXS). We show that the TRIM20 coiled-coil/B30.2 fragment, but not the isolated B30.2- and coiled-coil domains, binds pro-IL-1β, and we discuss different possible binding modes of TRIM5α, a close homologue of TRIM20, to the HIV-1 CA in light of the presented findings.

## Results

### Crystal Structure of TRIM20Δ413

**Figure 1:**
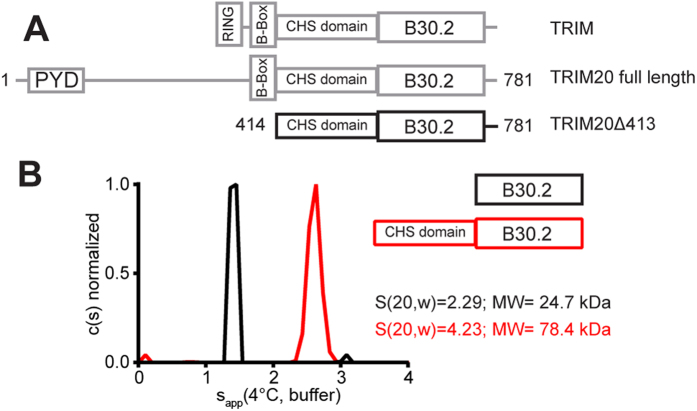
Central position of the CHS domain in TRIM proteins and its impact on dimerization. (***A***) Schematic representation of the domains of TRIM proteins comprising RING, B-Box, and CHS domains. In the literature the CHS domain is referred to as coiled-coil domain with a L2 linker that connects to the C-terminal B30.2 domain. TRIM20 is a RING-less TRIM member and has a long N-terminal extension composed of a PYD domain and a long linker region. For crystallization a construct starting at position 414 was designed. (***B***) Sedimentation velocity analytical ultracentrifugation data of the isolated B30.2 and CHS-B30.2 (TRIM20Δ413) fragments of TRIM20 show a monomer and a dimer, respectively. The molecular masses of monomers calculated from the B30.2 and CHS-B30.2 sequences are 23 305 Da and 42 737 Da, respectively.

To investigate the oligomerisation state and the spatial domain orientation of TRIM20 we designed a construct that comprises the TRIM20 coiled-coil and B30.2 domains. This design was guided by secondary structure prediction of TRIM proteins, which suggested that the bipartite coiled-coil motif starts immediately after the B-box domain. The alignment of human TRIM5α, −20, −21, −22, and −27 sequences revealed a conserved glutamic acid at position 414 (Fig. S1). Therefore, we engineered a construct starting with E414 yielding a fragment called TRIM20Δ413 (Fig. 1A). Analytical ultracentrifugation at 4 °C shows that this C-terminal fragment forms a dimer in solution, whereas the isolated TRIM20 B30.2 domain starting at T577^21^ forms a monomer (Fig. 1B).

**Figure 2:**
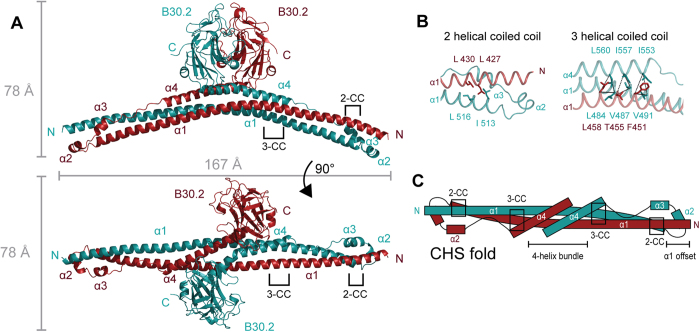
Crystal structure of TRIM20Δ413. The crystal structure of one out of three dimers from the asymmetric unit is shown. Chains A and B are colored in cyan and red, respectively. Helices of each CHS domain are numbered α1 to α4. The components of the CHS domain that participate in 2-helical (2-CC) or 3-helical coiled coils (3-CC) are indicated and depicted in more detail in *B*. (***A***) The dimer is shown from a side and a top view and its dimensions are indicated. (***B***) Residues involved in the 2-CC (top) and 3-CC (bottom) are shown as sticks and labeled in the corresponding color of the helix. (*C*) Topology of the CHS domain including its structural motifs are shown schematically.

TRIM20Δ413 crystallized in space group P2_1_ with 6 chains in the asymmetric unit (Table S1 and Fig. S2A). In the crystal TRIM20Δ413 forms a clear dimer with a coat-hanger-like shape. Dimerization is achieved by the coiled-coil motifs (E414–N586) that form an elongated and slightly bend scaffold for the globular B30.2 domain (E589–G776). The B30.2 domain is connected to the scaffold domain by a flexible loop (V587-P588) that acts as a hinge (Fig. 2A). Because residues E414–N586 serve as a scaffold to present the B30.2 domains we refer to this part of the structure as the central helical scaffold (CHS) domain. Each monomer of the CHS domain folds into 4 α-helices (α1-α4). Helix α1 (E414-E520) harbors the predicted coiled-coil motif and spans a length of approximately 155 Å (Fig. 2A). The designated L2 linker consists of two short helices α2 and α3 (E524–K539) followed by a proline-rich extended stretch (T540–P550) and helix α4 (Q551–N586). Helices α2 and α3, the proline-rich stretch and helix α4 are oriented antiparallel to helix α1, which is facilitated by a short hairpin loop between α1 and α2 (Q522–S523).

The subunits of the TRIM20Δ413 dimer arrange in an antiparallel fashion along helix α1 with a shared interface of more than 4700 Å^2^. This interface is created by all four helices including the extended stretch. As a result, the presumed B-box domains at the N-termini of TRIM20Δ413 are positioned approximately 170 Å apart from each other, whereas the B30.2 domains are close to the 2-fold axis of the dimer. Residues L473 from helix α1 and F574 from helix α4 are in direct proximity to the 2-fold dimer axis. The bipartite coiled-coil signature sequences (CC) are crucial features for the prediction of TRIM proteins. The 2-CC region participates in a 2-helix coiled-coil motif involving residues L427/L430 and residues L513’/L516’ at the N- and C-termini of helix α1, respectively (the prime indicates residues from the second protomer). The 3-CC region participates in a 3-helix coiled-coil motif involving residues L484/V487/V491, I553/I557/L560, and F451’/T455’/L458’ from helices α1, α4, and α1’, respectively (Fig. 2B and C). On the sequence level the 2-CC region is highly conserved in TRIM proteins, whereas the 3-CC seems to be TRIM20 specific with little sequence conservation among other TRIM proteins (Fig. S1). Furthermore helices α1, α1’, α4, and α4’ form a four-helix bundle at the core of the TRIM20Δ413 structure close to the 2-fold axis. From this core the B30.2 domains are pointing along the 2-fold axis and the helical extensions of the core including the L2 linker are slightly bent, reminiscent of a coat hanger.

**Figure 3:**
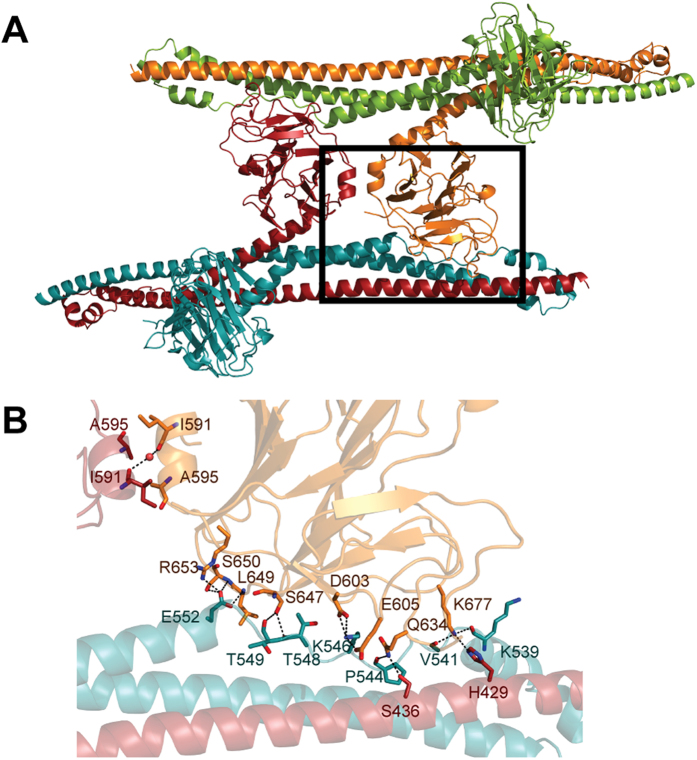
Interface between the B30.2 domain and the extended proline-rich stretch. (***A***) The crystal contact between two dimers is shown in a cartoon representation. Chains A and B are colored as in Fig. 2. The second dimer made of chain C and D is colored in orange and green, respectively. The black box indicates the display detail shown in *B*. The transition from the *closed* to the *open state* would require a swapping of the B30.2 domains shown in orange and red. (***B***) Detailed view of the B30.2/CHS domain interface. Residues involved in the interface are depicted in sticks and hydrogen bonds are indicated by black dashed lines. A water molecule between the B30.2 domains is shows as a red sphere.

Within the TRIM20Δ413 dimer the B30.2 domains neither interact with each other nor with the CHS domain, supporting the assumption that the orientation of the B30.2 domain might be highly flexible in solution. The curvature of the CHS domain and the orientations of the B30.2 domains differ among the three NCS related dimers (Fig. S2) suggesting that TRIM20 is sufficiently rigid to support lattice formation but still flexible enough to adopt to spatial constrains that are imposed by the structural environment. The fact that the TRIM20Δ413 structure doesn’t reveal an intra-molecular interface between the B30.2 and CHS domains is surprising (in this context intra-molecular refers to an interaction within the TRIM20Δ413 dimer), because this interaction was predicted by co-immuno precipitation experiments between isolated B30.2 and B-box/CHS constructs^8^. However, B30.2 and CHS domains share interfaces between neighboring dimers (Fig. 3A). These inter-molecular interfaces cover areas between 1710 and 1950 Å^2^ and are formed by the extended proline-rich stretch of the CHS domain that runs through a shallow surface cleft between the Pry- and Spry segments of the neighboring B30.2 domain (Fig. 3B). The B30.2/CHS domain interface harbors several hydrogen bonds and van der Waals interactions.

It is striking to see that helices α3 and α4 are separated by helix-breaking proline residues and that this extended stretch forms specific interactions with the B30.2 domain. Because of that and since it is also a structural feature in the coiled-coil domain of other TRIM or TRIM related proteins, we hypothesize that the interface between the B30.2 domain and the extended proline-rich stretch of the CHS domain might be functionally relevant. It could serve as an inter-molecular contact for TRIM oligomerization, but it could also be converted into an intra-molecular contact by a rearrangement of the B30.2/CHS domain linker. Depending on the linker conformation the B30.2 domain could adopt an *open state* where the B30.2 domain forms an intra-molecular contact to the CHS domain and a *closed state* with no B30.2/CHS domain interface.

### Solution structure of TRIM20Δ413

**Figure 4:**
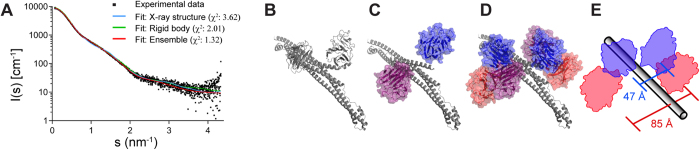
Solution structure of TRIM20Δ413. (***A***) Data extrapolated to infinite dilution is shown together with the theoretical curves calculated from the crystal structure, rigid body and ensemble optimized structures together with the χ^2^ values. (***B***) The crystal structure is shown in a cartoon representation next to the rigid body model (***C***), three representatives of the ensemble (***D***), and a schematic illustration of the distances between the B30.2 domains found in the crystal structure and the widest arrangement of the ensemble (***E***). The orientation found to be most similar to the crystal structure is shown in a blue surface representation. Other orientations of the B30.2 domain found in the rigid body and ensemble fit are colored violet and red.

In order to investigate the conformational flexibility of the B30.2 and CHS domains we performed small-angle X-ray scattering (SAXS) experiments. The radius of gyration (RG) for an infinitely diluted sample of TRIM20Δ413 is 4.27 nm, which is consistent with the RG calculated from the structure of the dimer in the *closed state* (4.29 nm; Fig. 4A). However, the measured RG value increases with protein concentration, suggesting that dimeric TRIM20Δ413 forms oligomers at elevated protein concentration (Fig. S3A to C). Furthermore, the crystal structure doesn’t fit exactly the solution scattering data in the scattering range *s* = 0.8 to 1.8 nm^−1^ (χ^2^ = 3.62) (Fig. 4A,B) and the Kratky-plot reveals inter-domain flexibility (Fig S3D). Therefore, a rigid body model was generated by introducing flexibility between the CHS- and the B30.2 domains. This model fits the experimental scattering data much better than the static model (χ^2^ = 2.01). In this model, one of the two B30.2 domains is arranged as in the crystal structure. The second B30.2 domain is rotated perpendicular to the 2-fold axis placing it sideways onto the CHS domain (Fig. 4A,C). Fitting an ensemble of structures improved the fit even further (χ^2^ = 1.31) and reveals the conformational space available for the B30.2 domain (Fig. 4A,D).

The conformation seen in the crystal structure defines one populated state in solution. In additional we see further states in solution where the B30.2 domains are positioned sideways onto the CHS domain. This movement increases the radius where the B30.2 domain could interact with its ligand. In the crystal structure the distance between the B30.2 centers of mass is about 47 Å. Movement of the B30.2 domain sideways increases this distance to 85 Å (Fig. 4E). Notably, a conformation of the B30.2 domain that would resemble an intra-molecular binding to the extended proline-rich stretch of the CHS domain was not observed in any model obtained from the SAXS data. The flexibility of the CHS domain, as suggested by the NCS of the crystal structure, was not modeled. Nevertheless, conformational flexibility of the CHS domain is also suggested by SAXS measurements as the average diameter found in solution is around 160 Å, which is a little less than in the crystal structure (167 Å).

### Pro-IL-1β Binding

**Figure 5:**
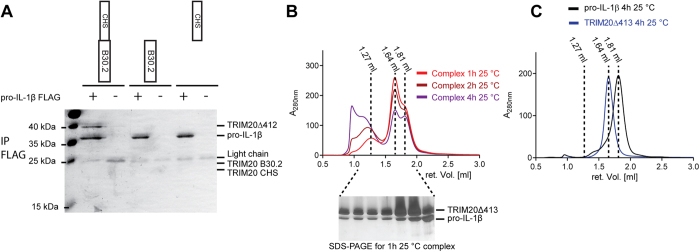
Interaction between TRIM20 and pro-IL-1β. (***A***) 10 μM of the TRIM20 constructs were incubated with 10 μg FLAG-tagged pro-IL-1β in a total volume on 100 μl. Complex was co-immunoprecipitated using ANTI-FLAG® M2 Affinity Gel (Sigma-Aldrich) and subjected to SDS-PAGE analysis. (***B***) TRIM20Δ413 and pro-IL-1β were incubated as indicated and their SEC profiles are shown together with the corresponding retention volumes of 1.27 ml, 1.64 ml, and 1.81 ml for the complex after 1 h incubation, TRIM20Δ413 and pro-IL-1β, respectively. Co-elution of both proteins was verified by SDS-PAGE of the indicated fractions. (***C***) Elution profiles of the individual proteins incubated for 4 h at 25° C show no signs of peak shifts.

Despite the large amount of data on the implication of TRIM20 in FMF, information on molecular interaction partners that trigger TRIM20 activation is sparse. Papin and colleagues revealed that full-length TRIM20 interacts with pro-IL-1β in a cell-culture system^8^. To investigate whether TRIM20 directly binds to pro-IL-1β *in vitro* we performed co-immuno precipitation experiments using purified pro-IL-1β and various constructs of TRIM20. These results show that TRIM20Δ413 directly binds to pro-IL-1β, whereas the isolated CHS- and B30.2 domains do not (Fig. 5A). To investigate the dissociation constant of this interaction we performed surface plasmon resonance (SPR) experiments using TRIM20Δ413 and pro-IL-1β. The data was fitted using the heterogeneous ligand model with apparent dissociation constants of 0.70 μM and 13.7 μM (Table S3 and Fig. S4A).

Complex formation between TRIM20Δ413 and pro-IL-1β was also detected by size exclusion chromatography (SEC). Stable complex formation however was only observed after incubating the proteins at 25 °C, whereas no complex formation was observed after incubation at 4 °C (Fig. 5B and Fig. S4B). The sample that was incubated at 25 °C showed a large peak at a retention volume of 1.27 ml corresponding to an apparent molecular weight of approximately 400 kDa (retention volumes: void, 0.8 mL; Trim20Δ413, 1.64 mL; pro-IL-1β, 1.81 mL). Prolonged incubation times at 25 °C yielded even higher oligomers (Fig. 5B). Nevertheless, complex formation was reversible. When the complex that was formed after 1 h incubation was re-injected, SEC revealed an equilibrium between the complex and the individual components (Fig. S4C). Neither TRIM20Δ413 nor pro-IL-1β alone showed signs of oligomerization upon incubation at 25 °C (Fig. 5C).

## Discussion

### Structural and functional properties of TRIM20

**Figure 6:**
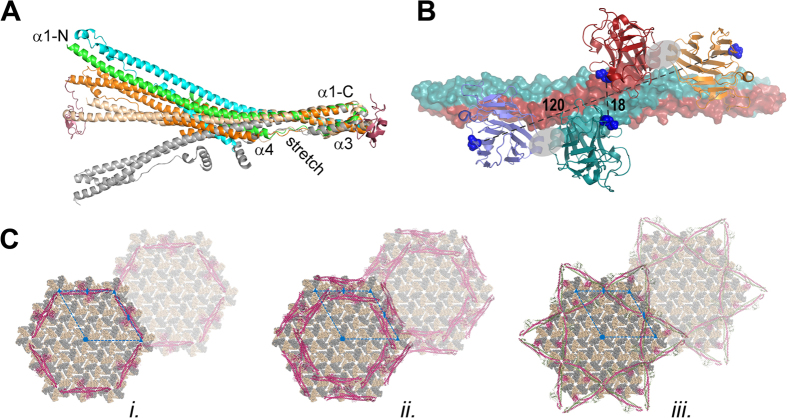
(A) Superposition of CHS domains of TRIM20Δ413 (chains C and D in cyan and green, respectively), TRIM5α B-box/coiled-coil (CHS-domain and B-box domains in salmon and red, respectively)^29^, TRIM25 coiled-coil (orange)^28^, and TRIM69 (grey)^27^. Helices α1 (N- and C-termini), α3, α4 and the extended stretch are labeled. (***B***) Illustration of *open*- and *closed states*. Crystal structure of TRIM20Δ413 (chains C and D are shown in red and cyan, and the B30.2 domains of chains B and E in orange and blue, respectively). B30.2 domains in orange/blue and red/cyan indicate the *open*- and *closed states*, respectively. The side chain of M694 from the putative peptide binding site of TRIM20 is shown as blue spheres. The hinge region for swapping between *open* and *closed states* is indicated by grey spheres. Distances between M694 residues are shown as dashed lines. (***C***) Three docking models for the binding of TRIM5α to HIV-1 CA. 19 CA hexamers (equivalent to 114 CA subunits) are shown as spheres in salmon and grey. TRIM5α is shown as ribbons. The asymmetric unit of the lattice is indicated by a blue dashed line (a = b = 187 Å, γ = 120°). Some 6-, 3- and 2-fold axes are shown as blue hexagons, triangles and ellipses, respectively. A second unit cell is shown in lighter colors. (i.) Model of the symmetric dimer in the *closed state*. (ii.) Model of the symmetric tetramer. (iii.) Model of the asymmetric dimer. Ribbons in magenta are experimental structures representing the *closed state*. Ribbons in green represent the *open state* that was modeled assuming a domain swapping of the B30.2 domain as described in the text.

Several crystal structures of isolated TRIM protein domains have been reported and the comparison of these TRIM fragments with the TRIM20Δ413 structure reveals valuable insight into the function of TRIM proteins Fig. 6A. A comparison of the isolated B30.2 domain structures between TRIM20, TRIM21/Fc^25^ and Ret finger protein-like 4A^26^ as well as mapping of FMF-associated mutations suggested that M694 of TRIM20 is involved in ligand binding^21^. However, in the TRIM20Δ413 dimer M694 is located close to the 2-fold axis and access to this putative peptide binding site is restricted by the B30.2 domain of the second protomer (Figs. 2A and 6B). Consequently, the B30.2 domains of TRIM20 have to move away from the 2-fold axis to recognize a ligand with a diameter of more than 20 Å. Therefore, we refer to the conformation seen in the crystal structure of TRIM20Δ413 as the *closed state*. The hypothesis that this state is flexible enough to be converted into an active conformation is supported by the solution scattering data and the topology of TRIM20Δ413. The best interpretation of the SAXS data is given by an ensemble of TRIM20Δ413 structures with B30.2 domains arranged in various conformations along the CHS domain. The topology enables this movement, because the B30.2 domain is not restricted by a stiff linker.

Interestingly the TRIM20Δ413 structure also suggests an *open state*, where the putative peptide binding sites are accessible even for very large ligands. The modelling of the *open state* relies on the assumption that the interaction between the proline-rich stretch and the B30.2 domain that is observed in the TRIM20Δ413 structure as an inter-molecular contact is converted into an intra-molecular contact by a rearrangement of the hinge region (Fig. 3A). In the *open state* the putative peptide binding sites would be directly accessible and a distance of approximately 120 Å between M694 residues would allow TRIM20 to bind to very large ligands with both sites simultaneously (Fig. 6B).

Structural and mutagenesis data support the hypothesis that the interface between the B30.2 domain and the extended proline-rich stretch has biological significance. The buried surface of this interface is extremely large (approximately 1800 Å^2^). It is almost three-times as large as the average surface area of a crystal contact and the biological significance of a protein/protein interface typically increases with its size^30^. In addition the extended – in the case of TRIM20 proline-rich – stretch is a conserved structural feature of TRIM proteins. The superposition of the TRIM20Δ413, TRIM25 coiled-coil^28^, TRIM69 coiled-coil^27^ and TRIM5α B-box/coiled-coil^29^ structures reveals that the curvatures of the CHS domains are extremely variable, but the topology of the helix bundle comprising the C-terminus of helix α1, the L2 linker, the extended stretch and the N-terminus of helix α1’ is conserved in all structures (Fig. 6A). The L2 linker is conserved, because it positions the B-box domain. Likewise, the insertion of an extended stretch between helices α3 and α4 can be explained by its role in either positioning the B30.2 domain in the *open state* of TRIM20 or the formation of a TRIM20 tetramer.

The mapping of mutations involved in FMF on the surface of the isolated TRIM20 B30.2 domain leads to the hypothesis that the B30.2 domain recognizes different ligands involving spatially separated binding sites^21^. This hypothesis is corroborated by the TRIM20Δ413 structure and in fact the biological significance of the B30.2/CHS domain interface is supported by the observation that mutations that are associated with mild forms of FMF, Crohn’s disease or arthritis, such as P550A^31^, L649P^32^, S650Y and R653H^33^, are directly involved in this interface (Fig. 3B). Mutations I591T^34^ and A595V are located in the linker between the CHS and B30.2 domains. These mutations could shift the equilibrium between the *open*- and *closed states* and thereby the affinity for the TRIM20 ligand.

On the other hand the arrangement of B30.2 domains as it is proposed for the *open state* was not observed among the ensemble of structures in the SAXS experiments. SAXS experiments suggested that TRIM20Δ413 has sufficient flexibility to reach this state, but the B30.2 domains never locked into a conformation seen in the B30.2/proline-rich stretch interface. The absence of this conformation from the ensemble of SAXS structures can be explained by the lack of a TRIM20 ligand. Perhaps the *open state* is only populated in a TRIM20/ligand complex. On the other hand TRIM20Δ413 formed higher order oligomers at elevated protein concentration in the SAXS measurements and the interaction between the B30.2 domain and the proline-rich stretch could explain this behavior.

The observation that isolated TRIM20 B30.2- and CHS domains were unable to bind pro-IL-1β confirms that dimerization of TRIM20 by means of the CHS domain is required for binding. The apparent molecular mass of 400 kDa suggests that the TRIM20Δ413/pro-IL-1β complex will most likely consist of several TRIM20Δ413 (85.7 kDa dimer) and pro-IL-1β (31.9 kDa) subunits and indicates that complex formation requires oligomerization of TRIM20Δ413 and/or pro-IL-1β. Additional experiments are required to reveal the exact stoichiometry of the TRIM20/pro-IL-1β complex. Complex formation also requires elevated temperature, because at 4 °C no interaction between TRIM20Δ413 and pro-IL-1β was detected. It is unlikely that the 400 kDa peak in the size exclusion chromatogram indicates temperature induced unfolding and unspecific aggregation, because the isolated components did not show any sign of aggregation at this temperature. The premise of elevated temperature could indicate that larger, temperature-induced structural rearrangements are required for binding.

### Modelling of TRIM interaction

Unfortunately neither the structure of pro-IL-1β nor the exact stoichiometry of this complex is known and attempts to crystallize it were so far unsuccessful. To exemplify how the CHS-domain, which has the same structural topology in many TRIM proteins (Fig. 6A), is involved in the recognition of higher order oligomers, we modelled the complex between TRIM5α and the HIV-1 CA lattice (pdb entry: 3dik)^35^ in a qualitative manner. TRIM20 differs from many other TRIM proteins including TRIM5α by a different domain composition. TRIM5α harbors a RING domain 30 amino acids N-terminal to the B-box 2 domain that was shown to participate in higher order self-association, whereas in TRIM20 a PYD domain is connected to the B-box 2 domain by a 60 or 280 amino acid linker. Since human TRIM5α and TRIM20 both comprise CHS- and B30.2 domains at the C-terminus of the B-box 2 domain, the TRIM20Δ413 structure is a suitable template to model the TRIM5α/CA interaction. Furthermore, the L2 region of TRIM5α was shown to contribute to self-assembly and CA recognition^36^ and mutations within the proline-rich stretch were found to abrogate HIV-1 restriction activity^37^,^38^. Mutations on the TRIM5α B30.2 domain that impair retroviral restriction activity but do not map to the predicted CA binding surface^39^,^40^ support the hypothesis that the interactions between the B30.2 domain and the proline-rich stretch also apply to TRIM5α.

We investigated two symmetric dimers with strict 2-fold symmetry (TRIM20Δ413 with both B30.2 domains in the same *open*- or *closed* conformation, Fig. 6B and model *i*. in Fig. 6C), a symmetric tetramer composed of chains C, D, E, and F from the TRIM20Δ413 crystal structure (model *ii*. in Fig. 6C) and an asymmetric dimer comprising a mixture of B30.2 domains in *open*- and *closed states* (model *iii*. in Fig. 6c). All three models (Fig. 6C) agree with mutagenesis data on TRIM5α/CA interaction^41^,^42^,^43^,^44^,^45^,^46^,^47^,^48^ and revealed lattice constants of 187 Å, which are in good agreement with the experimentally determined lattice constant of TRIM5α^35^. In the model of the symmetric dimer with the B30.2 domains in the *closed state* (model *i*.) the assumed peptide binding sites of B30.2 domains are not directly accessible. This model is almost identical to the models suggested by Sanchez *et al*. (2014) and Goldstone *et al*. (2014) that were build based on the structures of TRIM25 coiled-coil and TRIM5α B-box/coiled-coil domains, respectively^28^,^29^. However, the SAXS data on TRIM20Δ413 confirms that the B30.2 domains are sufficiently flexible to move into a conformation that allows direct recognition of HIV-1 CA. The main limitation of this model comes from the observation that the B-box 2 domains are predicted to form trimers although it was shown by NMR and analytical ultracentrifugation that the TRIM5α B-box 2 domains form dimers in solution^49^. The model of the symmetric dimer with the B30.2 domain in the *open state* (data not shown) has the same limitation. The docking of symmetric dimers suggests a stoichiometry of TRIM5α to CA of 1:12.

These limitations are eliminated if it is assumed that the TRIM lattice is composed of tetramers (model *ii*. in Fig. 6c). Here, the B30.2 domain serves a dual function. Two B30.2 domains build up the tetramer by binding to the extended stretch and the remaining two B30.2 domains interact with the CA. In this model the distance between the putative peptide binding sites of B30.2 domains fit exactly the diameter of the CA hexamer. Thus, two CA subunits can be recognized by two B30.2 domains simultaneously and the B-box 2 domains would form dimers. In the tetramer model B-box/B-box and B30.2/CHS domain interactions are required to build the TRIM lattice, but it was shown that lattice formation does not require the B30.2 domain^35^. Thus, the tetramer model can only be valid under the assumption that additional interactions between B-box 2 domains (besides dimerization) are responsible for lattice formation.

The third option would be an asymmetric dimer with one chain in the *open*- and the second chain in the *closed state* (model *iii*. in Fig. 6c). This model relies on the assumption that the inter-molecular interface between the B30.2 domain and the extended stretch is transformed into an intra-molecular interface by a rearrangement of the linker region (Fig. 6B). In this model the putative peptide binding sites of both B30.2 domains could directly interact with the CA epitope and the B-box 2 domains would form dimers. In this model the B30.2 only serves for capsid recognition. Therefore, elimination of the B30.2 domain does not prevent lattice formation. In this model two B-box 2 dimers line up along the six-fold axis of the hexamer (Fig. S5). Hence, additional interactions between B-box 2 dimers are also envisaged for this model. Furthermore, this model suggests weak electron density around the 3-fold axis, which disagrees with the projection density map of the TRIM5α/CA electron microscopy structure^35^. Models *ii*. and *iii*. suggest 1:6 stoichiometries for TRIM5α to CA. In summary, none of the models combines all experimental findings and all three models have their strengths and weaknesses. Therefore, further experimental evidence, e.g. the experimental analysis of the Trim5α/CA interaction stoichiometry, is needed to resolve these issues. It is hoped that the various models presented here would trigger efforts in this direction.

#### Experimental Procedure

Cloning, expression and purification, SAXS experiments, co-immuno precipitation experiments, surface Plasmon resonance analysis, size exclusion experiments and model building are described in the supplementary material file.

### Crystallization and structure determination

First crystals were found in 0.1 M Tris-AcOH, pH 8–8.5, 0.2–0.4% (w/v) Cystamine, 0.2 M LiSO_4_, 8% (w/v) PEG 20’000, 8% (w/v) PEG 550 MME using sitting drop vapor diffusion at 20 °C. TRIM20Δ413 (7.5 mg/ml) was mixed in a 1:1 ratio with mother liquor. The obtained crystals were used for micro-seeding in 0.1 M Tris-AcOH, pH 7.4–8.0, 0.26% (w/v) Cystamine, 0.2 M LiSO_4_, 8% (w/v) PEG 20’000, 8% (w/v) PEG 550 MME using sitting drop vapor diffusion at 20 °C. The protein sample was concentrated to 5 mg/ml and mixed in a 1:1 ratio. Crystals grew within 24 h and were harvested within 18 days. Prior to flash freezing in liquid nitrogen, crystals were soaked in the mother liquor supplemented with 20% (v/v) ethylene glycol. A native data set of a TRIM20Δ413 crystal was recorded at the X06SA beam line of the Swiss Light Source (Paul-Scherrer Institut, Villigen, Switzerland). The protein crystallized in P2_1_ with 6 molecules in the asymmetric unit forming 3 dimers. Data was indexed, integrated and scaled with XDS^50^ to a resolution of 2.4 Å. Molecular replacement was done with Phaser^51^ using the B30.2 domain of TRIM20 as search model (pdb entry: 2wl1). Model building and manual fitting was done in Coot^52^. Refinement was performed with Phenix^53^. In the final model, 98% of all residues are in the favored Ramachandran area and no outliers were detected.

## Additional Information

**How to cite this article**: Weinert, C. *et al*. Crystal structure of TRIM20 C-terminal coiled-coil/B30.2 fragment: implications for the recognition of higher order oligomers. *Sci. Rep*. **5**, 10819; doi: 10.1038/srep10819 (2015).

## Supplementary Material

Supplementary Information
