# Supplementary material for: Crystal structure of TRIM20 C-terminal coiled-coil/B30.2 fragment: implications for the recognition of higher order oligomers
Source: Sci Rep. 2015 Jun 4;5:10819. doi: 10.1038/srep10819 (PMC4455283; doi:10.1038/srep10819)
Supplement: Supplementary Information [file srep10819-s1.pdf]

Supporting Information

Crystal structure of TRIM20 C-terminal coiled-coil/B30.2 fragment: implications for the recognition of higher order oligomers.

Christopher Weinert, Damien Morger, Aleksandra Djekic, Markus G. Grütter, and Peer R.E. Mittl

Department of Biochemistry, University Zürich, Winterthurerstrasse 190, 8057 Zürich, Switzerland

Supporting Information Methods

Construction of Expression Plasmids

The multiple cloning site 1 (MCS1) of the transfer vector pFBDM [1] was modified to enable fragment exchange (FX) cloning [2]. The *Sap*I restriction site between the transposon element Tn7R and the Col-E1 origin of replication was removed using Quikchange mutagenesis (Invitrogen). A *Nde*I restriction site was inserted into the MCS1 by Quikchange. The FX-cassette of the pBXC3H vector [2] was used as template (incl. coding region for C-terminal His_{10}-tag and 3C cleavage site) and cloned into the modified pFBDM vector using *Nde*I and *Xba*I restriction sites yielding a pFX3CH vector. The *Nde*I restriction site was later removed. All TRIM20 constructs were cloned into the pFX3CH template vector. Primers and coding regions are stated in Table S4.

A similar procedure as for pFX3CH was applied to modify a pET28 (Novagen) vector. The *Sap*I site near the BR322 origin of replication was removed with Quikchange. The FX-cassette of pBXH3C (incl. coding region for N-terminal His_{10}-tag and 3C-cleavage site) was inserted using the *NdeI* and *XhoI* restriction yielding a pET28-FXH3C vector. Pro-IL-1β (codon optimized for *E.coli* expression) was cloned into the pET28-FXH3C vector using primers as stated in Table S4.

Expression and Purification

All TRIM constructs were expressed in *Sf*9 cells using the manufactures protocol (Invitrogen). Cells were harvested after 48-72 h expression at 27 °C. *Sf*9 cells expressing TRIM20Δ413 were lysed in lysis buffer (50 mM Tris-HCl, pH 8, 300 mM NaCl, 20 mM imidazole, DNAse I, RNase I, Benzoase, and EDTA-free complete inhibitor cocktail (Roche Diagnostics)) using an Emulsi-Flex C3 homogenizer (Avestin, Canada). Cell debris was centrifuged (1 h, 4 °C, 20’000 rpm) and supernatant applied to Protino Ni-NTA agarose (Machery Nagel). His-tag was cleaved by His-3C Protease, the sample dialysed in 50 mM Tris-HCl, pH 8, 150 mM NaCl, 10 mM imidazole, 0.5 mM TCEP, and reapplied to Ni-NTA agarose. Cleaved TRIM20Δ413 was dialyzed in low salt buffer (20 mM Tris-HCl, pH 8, 50 mM NaCl, 0.5 mM TCEP) and applied on a RESOURCE Q anion exchange column (GE Healthcare). The protein was eluted with 7.5% high salt buffer (20 mM Tris-HCl, pH 8, 1 M NaCl, 0.5 mM TCEP) and dialysed in 20 mM Tris-HCl, pH 8, 100 mM NaCl, 0.5 mM TCEP. The protein could be stored at 4 °C for up to two weeks, or it was frozen in liquid nitrogen and stored at -80 °C until further use.

All other TRIM constructs were lysed using 0.5% CHAPS in lysis buffer, and purified as described above. After His-tag removal the proteins were applied on a Superdex 200 (10/300 GL; GE Healthcare; 50 mM Tris-HCl, pH 8, 150 mM NaCl, 0.5 mM TCEP). Proteins were frozen in liquid nitrogen after addition of glycerol to a final concentration of 8.7% (v/v). For TRIM20-CHS 300 mM NaCl was used throughout the purification.

Pro-IL-1β was expressed in *E.coli* BL21 (DE3) strain. Cells were grown in LB-media at 30 °C to an OD_{600nm} of 0.8, cooled down at 4°C for 30 min and expression induced with 0.1 mM IPTG (OD_{600nm} of 1.0). After 15 h expression at 18 °C, cells were harvested (4°C, 20 min, 4000 rpm) and lysed in 50 mM Tris-HCl, pH 8, 150 mM NaCl, 10 mM imidazole, DNAse I, RNase I, lysozyme, 0.5 mM PMSF, EDTA-free complete inhibitor cocktail (Roche Diagnostics) using an Emulsi-Flex C3 homogenizer (Avestin, Canada). Lysate was cleared and supernatant applied to Ni-NTA agarose. His-tag was cleaved by His-3C Protease, the sample dialysed in 50 mM Tris-HCl, pH 8, 50 mM NaCl, 0.25 mM TCEP) and reapplied to Ni-NTA agarose. As a final purification step the sample was applied on a Superdex 200 in 50 mM Tris-HCl, pH 8, 50 mM NaCl, 0.5 mM TCEP). Fractions corresponding to monomeric pro-IL-1β were pooled and either used directly or frozen in liquid nitrogen after the addition of 8.7% glycerol and stored at -80 °C.

Co-IP experiments

10 µg of FLAG-pro-IL-1β were incubated with 10 µM TRIM20Δ412, TRIM20-B30.2 or TRIM20-CHS in a total volume of 100 µl (50 mM Tris-HCl, pH 7.4, 50 mM NaCl, 0.05% Tween 20). The mixture was incubated for 30 min at room temperature (RT) to allow complex formation. Putative complexes were co-immunoprecipitated using 20 µl ANTI-FLAG® M2 Affinity Gel (Sigma-Aldrich) for 10 min at RT. Beads were washed 3 times, and proteins eluted with 45 µl 2x SDS loading buffer. Eluted samples were analyzed by SDS-PAGE and visualized using silver staining [3].

Surface Plasmon Resonance

The interaction between TRIM20Δ412 and pro-IL-1β was measured on a ProteOn™ XPR 36 using a ProteOn™ GCL sensor chip (Bio Rad) equilibrated with running buffer (PBS, pH 7.5, 0.02% Tween 20). Monoclonal Anti-c-Myc IgG1 (20 µg/ml in 10 mM acetate, pH 5; Roche Diagnostics) was immobilized using amine coupling chemistry until the chip was saturated (6000-6400 RU). TRIM20Δ412 (1 µM) was applied to the immobilized antibody in running buffer and equilibrated

until a stable baseline was reached (550-600 RU TRIM20Δ412; ΔRU < 0.2 RU/min). In total 5 lanes were immobilized with TRIM20Δ412. The sixth lane was coated with antibody only and used as a reference for unspecific binding. Single shot kinetic was applied using a two-fold dilution series of pro-IL-1β in running buffer (1.56, 3.125, 6.25, 12.5, 25 μM). For each lane two single shot experiments were performed. Data was double referenced against buffer and the antibody control lane. Binding affinity (K_D) was determined kinetically for each experiment using the heterogenous ligand model implemented in the ProteOn™-Software (Bio Rad). The mean and standard deviation of the measured K_Ds was determined using GraphPad Prism6 (GraphPad Software).

Size exclusion chromatography analysis of complex formation

TRIM20Δ413 was mixed with freshly prepared pro-IL-1β. 3 mg of each protein were mixed and concentrated to a final concentration of 19 mg/ml. The buffer was exchanged to 20 mM Tris-HCl, pH 8, 100 mM NaCl, 0.5 mM TCEP while concentrating. Individual proteins were concentrated to 8 mg/ml and buffer was exchanged as for the complex. Samples were incubated for 1, 2 and 4 h at room temperature or 20 h at 4 °C, centrifuged (14’000 g, 10 min, 4 °C) and 5 μl injected onto a Superdex 200 (5/150 GL, GE Healthcare) in 20 mM Tris-HCl, pH 8, 100 mM NaCl, 0.5 mM TCEP at 4 °C. 50 μl fractions were collected, analyzed by SDS-PAGE and visualized using silver staining.

Small-angle X-ray scattering

SAXS data were collected at the Petra III (P62) beam line at the Deutsches Elektronen Synchroton (DESY), Hamburg. The incident wavelength was 1.24 Å. Data was recorded on a 2M Pilatus detector for the angular range of s between 0.0668-4.336 nm^{-1} ($s=4\pi \sin(\theta)/\lambda$, where 2θ is the diffraction angle and λ the wavelength of the incident beam). TRIM20Δ413 was measured at 10 °C in PBS and 5 mM DTT at several concentrations (1.7, 2.88, 4.6, 8.75, 13.3 mg/ml). Data was processed with PRIMUS [4] and a scattering curve extrapolated to infinite dilution was created using ALMERGE [5]. The pair distribution function (P (r)) was calculated with GNOM [6]. Radius of gyration R_G was estimated from the Guinier plot using PRIMUS and the P (r) function using GNOM. Rigid body modeling was carried out in CORAL[7] defining residues 586-590 as a flexible linker. Modeling various conformations simultaneously was performed with the ensemble optimization method (EOM; [8]) using residues 588-589 as flexible linker between the domains. Nine different conformations of the B30.2 domain relative to the CHS domain were found by EOM analysis. Model with similar orientations of the B30.2 domain were combined in case their center

of mass was within 6Å. Comparison with the crystal structure and the experimental scattering profile was done with CRYSOL[9].

Sequence alignment

Sequences used correspond to the UniProt entries O15553, Q9C035, P19474, Q8IYM9, P14373 for TRIM20, TRIM5α, TRIM21, TRIM22, TRIM27, respectively. Alignments were generated using T-coffee [10] and Jalview [11]. Coiled coil predictions are taken from secondary structure predictions using Jpred 3 [12].

Modeling

To derive a hypothesis how TRIM5α could bind to the capsid (CA) lattice of HIV-1 the structure of TRIM20Δ413 was docked manually onto the 9 Å resolution structure of CA (PDB ID: 3dik) [13] using the program COOT [14]. Because the precision of this model is limited by the resolution of the CA, which was determined by electron microscopy, no attempts were made to generate a homology model of TRIM5α based on the experimental TRIM20Δ413 structure. TRIM5α (Uniprot ID: Q9C035) and TRIM20Δ413 share 25.8% sequence identity and the structures of isolated TRIM5α B30.2- and CHS domains (pdb entries: 3uv9 and 4tn3) are similar to TRIM20Δ413. Mutagenesis data confirm that residues 328-332 of the human TRIM5α B30.2 domain are involved in binding the HIV-1 CA [15]. Residues I328 and R332 from TRIM5α correspond to I612 and L622 from TRIM20Δ413. The epitope on CA comprises residues N82, E92, A95 and H114 [16-18]. These experimental results were used as restraints to dock the structure of TRIM20Δ413 in three different arrangements on the electron microscopy structure of HIV-1 CA: (i) two symmetric dimers either in the *closed*- (chains C and D) or *open states* (residues 413 to 595 from chains C and D and residues 596 to 772 from chains B and E), (ii) a symmetric tetramer (chains C, D, E, and F), and (iii) a mixed *open/closed state* dimer (residues 413 to 772 from chain C, residues 413 to 595 from chain D and residues 596 to 772 from chain E).

Model building was guided by the assumption of a lattice match between TRIM and CA lattices. Symmetric structures, such as the *open*- and *closed state* dimers or the tetramer, were aligned such that the 2-fold axis of TRIM20Δ413 and CA were collinear. Alternatively the 2-fold TRIM20Δ413 axis was aligned with the 6-fold CA axis, because a 6-fold axis is equivalent to parallel 2- and 3-fold axes. TRIM20Δ413 was than rotated and translated along the 2-fold axis until the epitope on the TRIM20 B30.2 domain matched the epitope on the CA. The epitope on the CA is defined by

residues 92 to 95 [17] and the epitope on TRIM20 by the putative peptide binding site around M694 [19] and by I612, because the replacement of the equivalent residue in TRIM5α (I328) abrogated the recognition of CA [15]. The asymmetric model for the *open/closed state* was docked as follows. Starting from the coordinates of CA (PDB ID: 3dik) we generated a CA hexamer by applying the crystallographic symmetry operators. The subunits of the hexamer are termed CA1, CA2 and CA3 (related by 3-fold symmetry) and CA1*, CA2*, and CA3*. CA1/2/3 are related to CA1/2/3* by 2-fold symmetry. A second hexamer was generated by applying a translation of one unit cell (a = 92.7 Å). The subunits of the translated hexamer are indicated by $^{\#}$. Residue M694 from the B30.2 domain in the *open state* was docked onto residue A95 from CA1 and M694' from the B30.2 domain in the *closed state* on residue A95 from CA2*$^{\#}$. This combination was chosen, because the vectors that relate the epitopes on TRIM (vector between M694 and M694' of 70 Å) and CA (vector between A95^{1} and A95$^{2*\#}$ of 57 Å) are most similar in length. The orientation of TRIM20Δ413 in the mixed *open/closed state* was than propagated by applying the 2-fold rotation of the untranslated hexamer (e.g. A95 of CA1' interacts with M694 in the rotated B30.2 domain of the *open state*) and applying the 3-fold rotation of the translated hexamer (e.g. CA1*$^{\#}$ and CA3*$^{\#}$ interact with M694' in the rotated B30.2 domains of the *closed states*).

Analytical ultracentrifugation

The B30.2 domain, expressed and purified as described [19], and TRIM20Δ413 were measured in PBS (pH 7.4) 10 mM β-Mercaptoethanol at a concentration of 0.6 mg/ml and 0.8 mg/ml, respectively. The sedimentation velocity measurements were carried out at 4°C on a ProteomeLab XL-1 (Beckman Coulter) ultracentrifuge at a speed of 30000 rpm. Data was analyzed with SEDFIT [20].

References

1 Berger, I., Fitzgerald, D. J. & Richmond, T. J. Baculovirus expression system for heterologous multiprotein complexes. *Nat. Biotech.* **22**, 1583-1587 (2004).

2 Geertsma, E. R. & Dutzler, R. A versatile and efficient high-throughput cloning tool for structural biology. *Biochemistry* **50**, 3272-3278 (2011).

3 Nesterenko, M. V., Tilley, M. & Upton, S. J. A simple modification of Blum's silver stain method allows for 30 minute detection of proteins in polyacrylamide gels. *J. Biochem. Biophys. Meth.* **28**, 239-242 (1994).

4 Konarev, P. V., Volkov, V. V., Sokolova, A. V., Koch, M. H. J. & Svergun, D. I. PRIMUS: a Windows PC-based system for small-angle scattering data analysis. *J. Appl. Cryst.* **36**, 1277-1282 (2003).

5 Franke, D., Kikhney, A. G. & Svergun, D. I. Automated acquisition and analysis of small angle X-ray scattering data. *Nuclear Instruments and Methods in Physics Research Section A: Accelerators, Spectrometers, Detectors and Associated Equipment* **689**, 52-59 (2012).

6 Svergun, D. Determination of the regularization parameter in indirect-transform methods using perceptual criteria. *J. Appl. Cryst.* **25**, 495-503 (1992).

7 Petoukhov, M. V. *et al.* New developments in the ATSAS program package for small-angle scattering data analysis. *J. Appl. Cryst.* **45**, 342-350 (2012).

8 Bernadó, P., Mylonas, E., Petoukhov, M. V., Blackledge, M. & Svergun, D. I. Structural Characterization of Flexible Proteins Using Small-Angle X-ray Scattering. *J. Am. Chem. Soc.* **129**, 5656-5664 (2007).

9 Svergun, D., Barberato, C. & Koch, M. H. J. CRYSOL– a Program to Evaluate X-ray Solution Scattering of Biological Macromolecules from Atomic Coordinates. *J. Appl. Cryst.* **28**, 768-773 (1995).

10 Keller, O., Kollmar, M., Stanke, M. & Waack, S. A novel hybrid gene prediction method employing protein multiple sequence alignments. *Bioinformatics* **27**, 757-763 (2011).

11 Waterhouse, A. M., Procter, J. B., Martin, D. M., Clamp, M. & Barton, G. J. Jalview Version 2--a multiple sequence alignment editor and analysis workbench. *Bioinformatics* **25**, 1189-1191 (2009).

12 Cole, C., Barber, J. D. & Barton, G. J. The Jpred 3 secondary structure prediction server. *Nucleic Acids Res.* **36**, W197-201 (2008).

13 Ganser-Pornillos, B. K. *et al.* Hexagonal assembly of a restricting TRIM5alpha protein. *Proc. Natl. Acad. Sci. U S A* **108**, 534-539 (2011).

14 Emsley, P. & Cowtan, K. Coot: model-building tools for molecular graphics. *Acta Crystallogr. D Biol. Crystallogr.* **60**, 2126-2132 (2004).

15 Stremlau, M., Perron, M., Welikala, S. & Sodroski, J. Species-specific variation in the B30.2(SPRY) domain of TRIM5alpha determines the potency of human immunodeficiency virus restriction. *J. Virol.* **79**, 3139-3145 (2005).

16 Mortuza, G. B. *et al.* Structure of B-MLV capsid amino-terminal domain reveals key features of viral tropism, gag assembly and core formation. *J. Mol. Biol.* **376**, 1493-1508 (2008).

17 Stevens, A. *et al.* Retroviral capsid determinants of Fv1 NB and NR tropism. *J. Virol.* **78**, 9592-9598 (2004).

18 Ohkura, S. *et al.* Novel escape mutants suggest an extensive TRIM5alpha binding site spanning the entire outer surface of the murine leukemia virus capsid protein. *PLoS Pathog.* **7**, e1002011 (2011).

19 Weinert, C., Grütter, C., Roschitzki-Voser, H., Mittl, P. R. & Grütter, M. G. The crystal structure of human pyrin b30.2 domain: implications for mutations associated with familial Mediterranean fever. *J. Mol. Biol.* **394**, 226-236 (2009).

20 Schuck, P. Size-distribution analysis of macromolecules by sedimentation velocity ultracentrifugation and lamm equation modeling. *Biophys. J.* **78**, 1606-1619 (2000).

21 Krissinel, E. & Henrick, K. Secondary-structure matching (SSM), a new tool for fast protein structure alignment in three dimensions. *Acta Crystallogr. D Biol. Crystallogr.* **60**, 2256-2268 (2004).

22 Fischer, H., de Oliveira Neto, M., Napolitano, H. B., Polikarpov, I. & Craievich, A. F. Determination of the molecular weight of proteins in solution from a single small-angle X-ray scattering measurement on a relative scale. *J. Appl. Cryst.* **43**, 101-109 (2010).

Figures

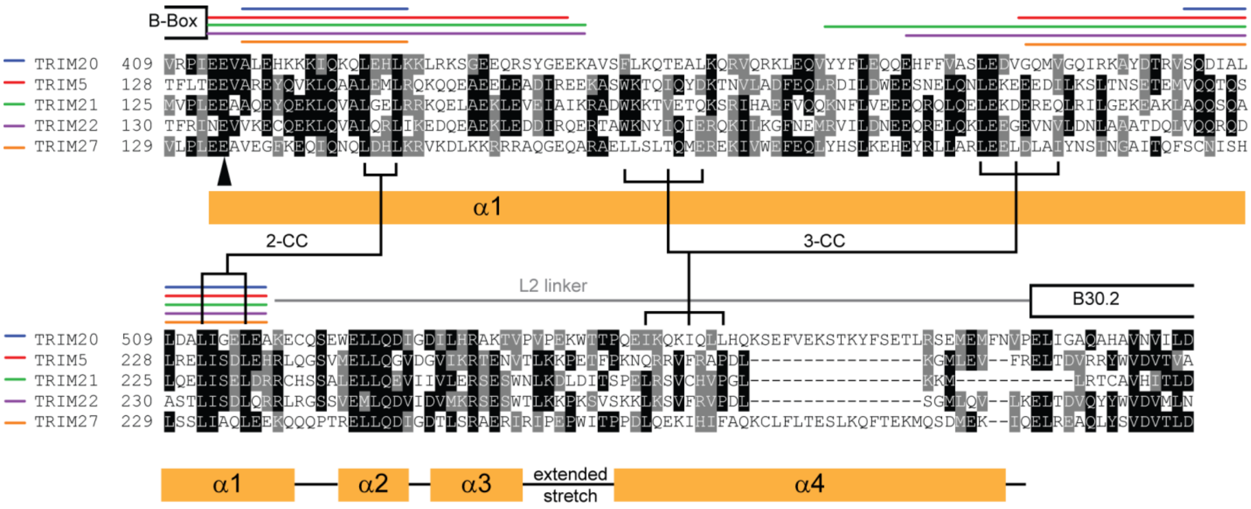

Figure S1. Sequence alignment of the CHS domain of several TRIM20 homologues. Identical and similar residues are depicted by black and grey boxes, respectively. The predicted bipartite coiled coils for every TRIM are indicated by lines on top of the sequences and colored as indicated next to the protein names. The predicted L2 linker is marked by the grey line. Secondary structure elements as seen in the TRIM20 structure are indicated below the sequences. Residues that form the 2 helical coiled coil (2-CC) and 3 helical coil coiled (3-CC) are connected by black lines.

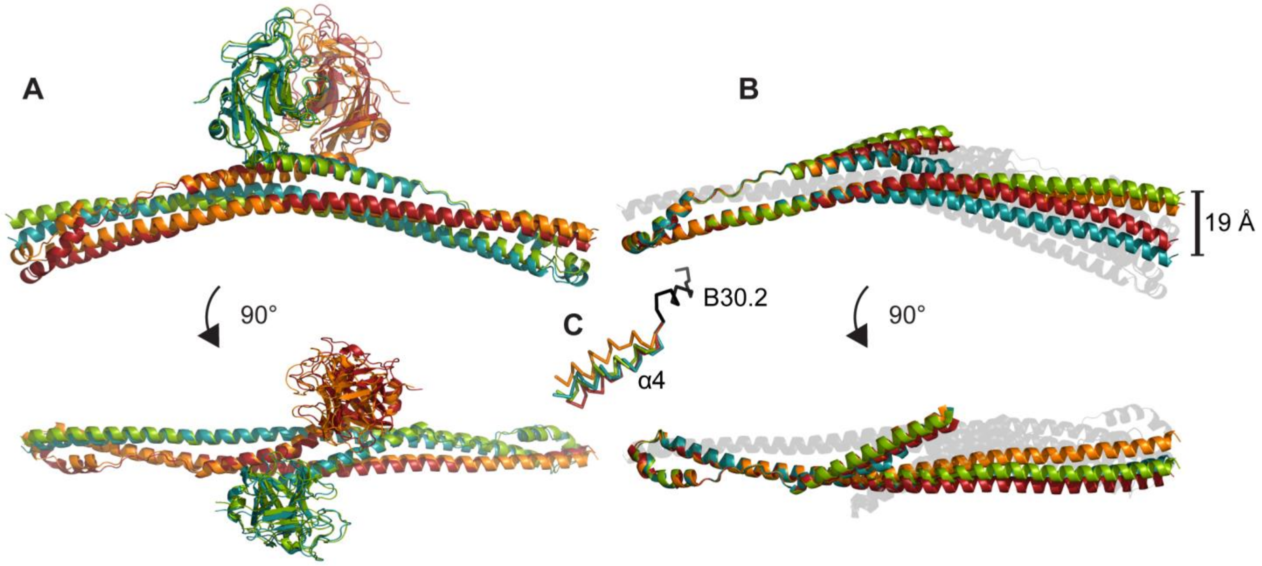

Figure S2. Different dimers in the asymmetric unit. Three dimers are found in the asymmetric unit (chains A&B, C&D, and E&F). For visualization only dimer of chain A&B and C&D were used. (*A*) Superposition of the TRIM20 dimers are shown in a top and side view. The chains are colored in blue, red, orange and green for monomer A, B, C and D, respectively. (*B*) For a better visualization of the differences between the CHS monomers residues 486-560 of each monomer were superposed using Superpose [21]. For each monomer the corresponding second protomer is shown in transparent black. (*C*) The hinge region between the helix α4 and the B30.2 domain is visualized by superposing the first N-terminal helix of each B30.2 domain (residues 590-595, shown in black). Helix α4 of each monomer is colored as in *A* and *B*.

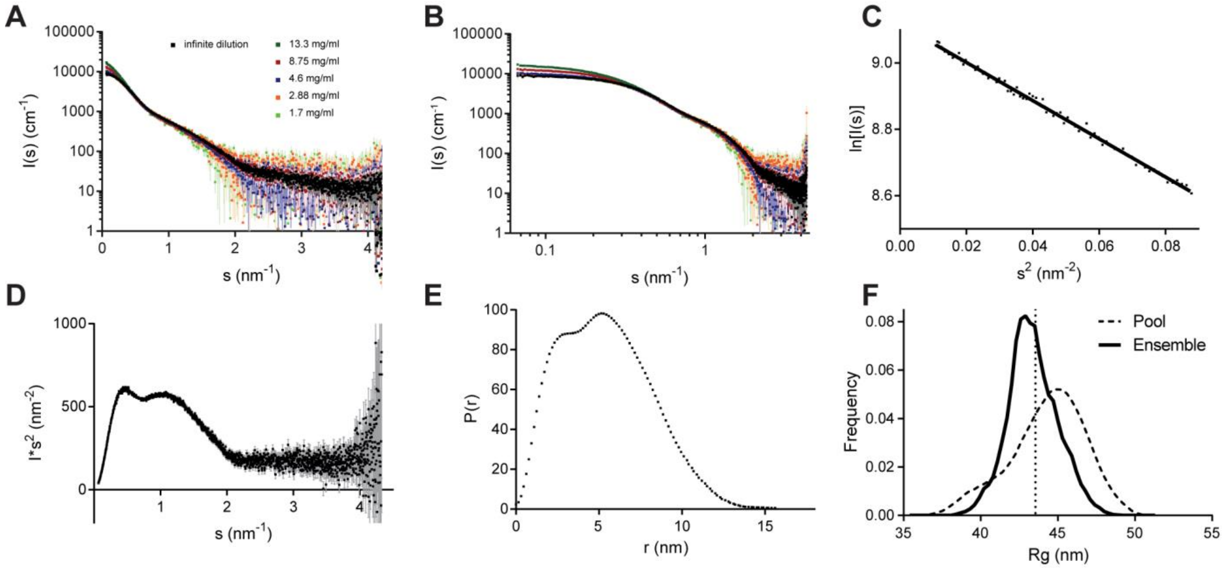

Figure S3. SAXS data. (*A*) and (*B*) Overlay of experimental scattering curves are shown for all measured concentrations as indicated. For structural modeling a dataset was created corresponding to an infinite dilution. In (*A*) and (*B*) the data is plotted as X-log Y and log X-log Y, respectively, with the error bars depicted as lines in the corresponding color. (*C*) For the dataset extrapolated to infinite dilution (black dots) the Guinier plot (black line) shows linearity without signs of inter-particle attraction. (*D*) The Krakty-plot (black dots) shows a typical curve for a two domain protein with inter-domain flexibility. The errors are depicted by grey lines. (*E*) The P(r) curve approaches zero for $r = r_{max}$. For $r = 0$, P(r) does not fully approach zero suggesting a minimal mismatch of the buffers in the sample and in the buffer control. (*F*) R_G distribution for the pool used in the ensemble optimization method and the selected ensemble. The vertical dashed line indicates the average R_G for the selected ensemble.

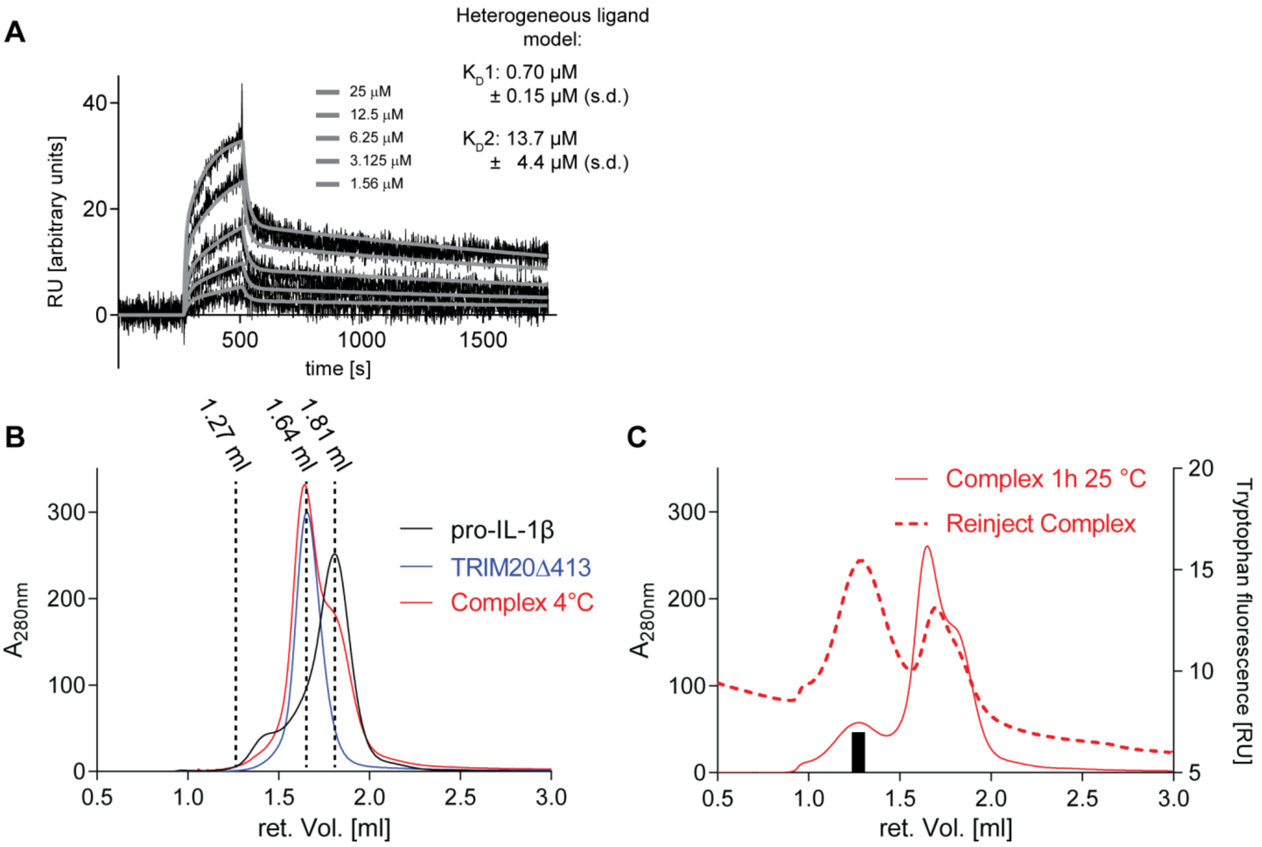

Figure S4. Binding of TRIM20 to pro-IL-1β. (*A*) Typical sensogram of the SPR measurements is shown. Myc-TRIM20Δ412 was immobilized and a dilution series of pro-IL-1β, as indicated, was subjected (black lines). The obtained fit for every concentration using the heterogeneous ligand model is shown by the grey lines and the resulting K_Ds and their standard deviations (s.d.) are stated. (*B*) SEC profile for pro-IL-1β, TRIM20Δ413 and both proteins incubated for 20 h at 4 °C (complex) are shown in the corresponding color. The dotted lines indicate the retention volumes for the individual proteins and the complex as seen in Fig. 5*B*. (*C*) SEC profile of a mixture of both proteins incubated as stated (complex) is shown (left y-axis). The black box indicates the fraction that was re-injected (re-inject complex; right y-axis).

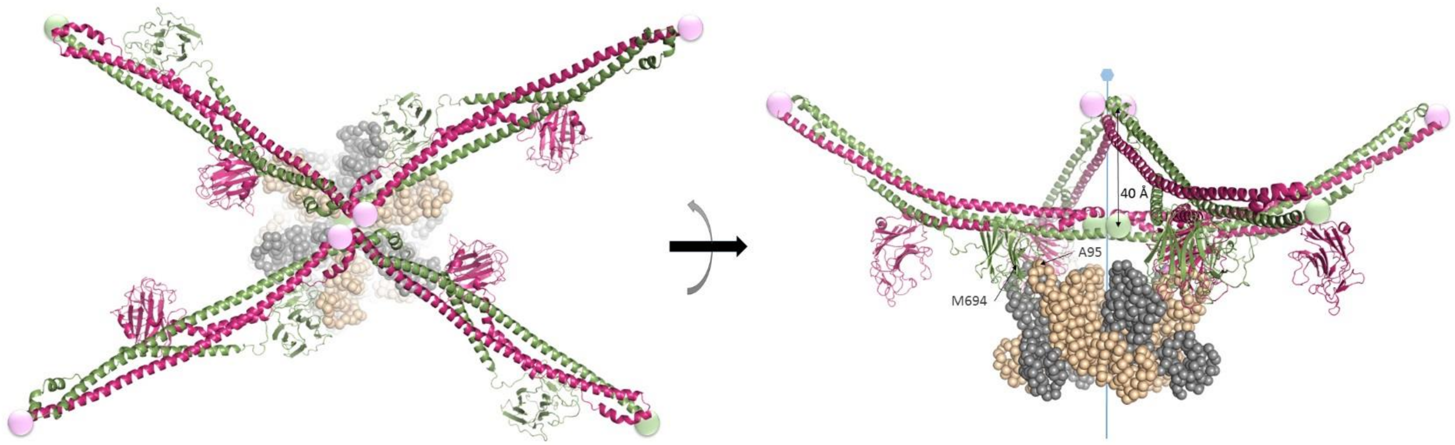

Figure S5. Perpendicular close-up views of the asymmetric dimer TRIM5α/CA interaction model. Only four TRIM5α dimers and one CA hexamer are shown using the same color scheme as Fig. 6C. In addition the B-box domains are sketched as pink (*closed state*) and green balls (*open state*). The 6-fold symmetry axis is shown as a blue line and the distance between N-termini of *open*- and *closed states* is shown. Residues A95 and M694, which indicate the epitopes on CA and the B30.2 domain, are labeled.

Supplementary Tables

Table S1. X-ray data collection and refinement statistics

	TRIM20Δ413
**Data collection**	
Space group	$P2_1$
Cell dimensions	
a, *b*, *c* (Å)	69.82 388.21 70.88
α, β, γ (°)	90 116.52 90
Resolution (Å)	48.53 - 2.4 (2.486 - 2.4)*
R_{merge}	0.1724 (1.857)*
$I / \sigma I$	9.77 (0.88)*
Completeness (%)	98.96 (93.07)*
Redundancy	7.4 (6.6)*
$CC_{1/2}$	99.6 (51.3)*
**Refinement**	
Resolution (Å)	48.53 – 2.4
No. reflections	129414
R_{work} / R_{free} (%)	21.15 / 26.07
No. atoms	
Protein	17943
Ligand/ion	68
Water	33
B-factors	
Protein	54.8
Ligand/ion	67.7
Water	33.6
R.m.s. deviations	
Bond lengths (Å)	0.012
Bond angles (°)	1.37

The dataset was recorded from a single crystal.
*Values in parentheses are for highest-resolution shell.

Table S2: SAXS data collection and processing

	TRIM20Δ413
**Data collection**	
Instrument	Petra 12; Pilatus 2M
Beam geometry	0.2x0.12 mm2
Wavelength (Å)	1.24
s range (nm^{-1})	0.0668-4.336
Exposure time (s)	0.05
Concentration range [mg/ml]	1.7-13.3
Temperature	283
**Structural parameters**	
I(0) (cm-1) [from P(r)]	9184 (± 8.47)
Rg (nm) [from P(r)]	4.27 (± 0.056)
I(0) (cm-1) (from guinier)	9100.84 (±20.24)
Rg (nm) (from guinier)	4.15 (± 0.023)
Dmax [nm]	15.9
Porod volume estimate (Å3)	131.72
Dry volume calculated from structure (Å3)	100.5
**Molecular mass determination**	
Partial specific volume (cm3g-1)	1.37
Molecular mass Mr [kDa][22]	90.0
Molecular mass Mr [kDa; analytical ultracentrifugation]	78.4
Monomeric Mr from MS analysis [kDa]	42.737
**Software employed**	
Primary data reduction	*PRIMUS*
Data processing	*PRIMUS / GNOM*
Extrapolation to infinite dilution	*ALMERGE*
Ab initio analysis	*DAMMIN*
Validation and averaging	*DAMAVER*
Rigid-body modelling	*SASREF*
Multiple conformation modelling	*EOM*
Computation of model intensities	*CRYSOL*
3D graphics representation R.m.s. deviations	*PyMOL*

Table S3. Kinetic data of SPR experiment

k_{on}1 [1/Ms]	k_{off} 1 [1/s]	K_D1 [µM]	R_{max}1 [RU]	k_{on}2 [1/Ms]	k_{off} 2 [1/s]	K_D2 [µM]	R_{max}2 [RU]	χ^2 [RU]
$4.17*10^2$ $\pm1.4*10^2$	$2.83*10^{-4}$ $\pm0.59*10^{-4}$	0.70 ± 0.15	20.3 ± 3.88	$3.96*10^3$ $\pm1.32*10^3$	$5.10*10^{-2}$ $\pm1.38*10^{-2}$	13.7 ± 4.4	25 ± 3.27	2.55 ±0.54

Table S4. Construct design

Construct	Coding region	Tag	Forward primer	Reverse primer	Template vector
TRIM20Δ413	414-781	-	atatatgctcttctagtgaggtcgccctggaac	tatatagctcttcttgcgtcaggcccctgacc	pFX3CH
TRIM20Δ412	413-781	N-terminal c-Myc (EQGKLISEEDL)	atatatgctcttctagtgagcagaaactcatctct gaagaggatctggaggaggttgccc	tatatagctcttcttgcgtcaggcccctgacc	pFX3CH
TRIM20-CHS	413-577	N-terminal c-Myc (EQGKLISEEDL)	atatatgctcttctagtgagcagaaactcatctct gaagaggatctggaggaggttgccc	tatatagctcttcatgcggtttctgagaagtac	pFX3CH
TRIM20-B30.2	577-781	C-terminal c-Myc (EQGKLISEEDL)	atatatgctcttctagtctgcgttcagaaatgg	tatatagctcttcatgccagatcctcttcagagat gagtttctgctcgtcaggcccctgacc	pFX3CH
Pro-IL-1β	Full length	C-terminal FLAG (DYKDDDDK)	atatatgctcttctagtgcggaagtgccggaac	tatatagctcttcatgc cttatcgtcgtcatccttgta atcgctgctcacaaactgc	pET28-FXH3C
